# Prioritization of Diagnostic and Prognostic Biomarkers for Lupus Nephritis Based on Integrated Bioinformatics Analyses

**DOI:** 10.3389/fbioe.2021.717234

**Published:** 2021-10-08

**Authors:** Zhimin Chen, Ruilong Lan, Keng Ye, Hong Chen, Caiming Chen, Yanfang Xu

**Affiliations:** ^1^ Department of Nephrology, Blood Purification Research Center, First Affiliated Hospital of Fujian Medical University, Fuzhou, China; ^2^ Central Laboratory, First Affiliated Hospital of Fujian Medical University, Fuzhou, China; ^3^ Department of Pathology, First Affiliated Hospital of Fujian Medical University, Fuzhou, China

**Keywords:** lupus nephritis, bioinformatics, biomarkers, diagnosis, prognosis, WGCNA

## Abstract

Lupus nephritis (LN) is an important driver of end-stage renal disease (ESRD). However, few biomarkers are available for evaluating the diagnosis and prognosis of LN. For this study, we downloaded microarray data of multiple LN expression profiles from the GEO database. We used the WGCNA and R limma packages to identify LN hub genes and differentially-expressed genes (DEGs). We identified nine co-DEGs in the intersection with LN-related genes from the Genecards database. We found DEGs that are primarily associated with immune-related functions and pathways (including with the complement pathway, primary immunodeficiency markers, and MHC-like protein complexes) through our comprehensive GSEA, GO, and KEGG enrichment analyses. We used other LN and SLE validation datasets and discovered six explicitly expressed co-DEGs: *HLA-DMA*, *HLA-DPA1*, *HLA-DPB1*, *HLA-DRA*, *IL10RA*, and *IRF8* in the LN set; ROC and Precision-Recall curve analyses revealed that these six genes have a good diagnostic efficacy. The correlation analysis with prognostic data from the Nephroseq database indicates that the differential expression of these co-DEGs is associated with a low glomerular filtration rate in that cohort. Additionally, we used a single-cell LN database of immune cells (for the first time) and discovered these co-DEGs to be predominantly distributed in different types of macrophages and B cells. In conclusion, by integrating multiple approaches for DEGs discovery, we identified six valuable biomarkers that are strongly correlated with the diagnosis and prognosis of LN. These markers can help clarify the pathogenesis and improve the clinical management of LN.

## Introduction

Systemic lupus erythematosus (SLE) is a common chronic autoimmune disease with multifactorial causes. SLE affects mainly women of childbearing age and its progression and prognosis are highly heterogeneous. The characteristics of SLE include the production of autoantibodies, the deposition of immune complexes, and impairment of multiple organ systems (Kiriakidou and Ching, 2020). A genetic predisposition, environmental factors, apoptosis abnormalities, infections, the use of certain drugs, and sex hormone levels are factors thought to play a role in the pathogenesis of SLE (Durcan et al., 2019). The kidney is the most commonly affected organ in patients with SLE, and renal biopsies show a nearly 100% involvement with approximately 45–85% of patients presenting clinical symptoms of lupus nephritis (LN) (Furie et al., 2020). The pathogenesis of LN includes a process of kidney damage caused by immune complex deposition in renal tissues (Anders et al., 2020) due to inflammatory cell recruitment, cytokine production, oxidative damage, complement activation, and abnormal fibroblast proliferation (Davidson et al., 2019). Inflammation and fibrosis are critical for the development of LN because they include the interaction of innate and adaptive immune cells with resident renal cells. Although glucocorticoids and immunosuppressants have been shown to improve survival in patients with LN, the current treatment for this disease remains unsatisfactory (Lech and Anders, 2013). Additionally, the adverse effects of non-specific treatments (including those against infection and renal failure) make a more effective and targeted approach urgent. Thus, conducting additional research into the etiology and pathogenesis of the disease is necessary to further improve the survival of patients with LN.

Bioinformatics is a branch of computer science that focuses on the storage, retrieval, and analysis of biological data. The analysis of massive amounts of data generated by biochips has provided helpful information to help understand molecular disease mechanisms (Wooller et al., 2017). Bioinformatics has been widely used to obtain disease gene expression profiles, to identify disease-related genes and drug targets, and to analyze complex disease pathogenic mechanisms. Craciun *et al.*, for example, used RNA sequencing to characterize the renal transcriptomic profile of specimens in a mouse model of folic acid-induced nephropathy (Craciun et al., 2016). As a result, they identified 10 molecules associated with renal fibrosis, with the levels of *CDH11*, *MRC1*, and *PLTP* being significantly increased in the urine of patients with chronic kidney disease. Köttgen *et al.* used genetic and genotype-population analyses to obtain 67093 European genome-wide SNPs and then performed a GWAS analysis to identify SNP mutations in *UMOD*, a susceptibility gene for chronic kidney disease (Köttgen et al., 2010). Additionally, GWAS studies have helped identify susceptibility genes for diabetic nephropathy and IgA nephropathy (Salem et al., 2019; Sallustio et al., 2019).

In this study, we used bioinformatics approaches to screen for co-differentially-expressed genes (co-DEGs) in LN from multiple LN dataset sources. The purpose of this study was to identify and prioritize diagnostic and prognostic biomarkers for lupus nephritis and to explore the potential pathways and immune cells that are related to the pathogenesis of LN.

## Materials and Methods

### Data Download

**FIGURE 1:**
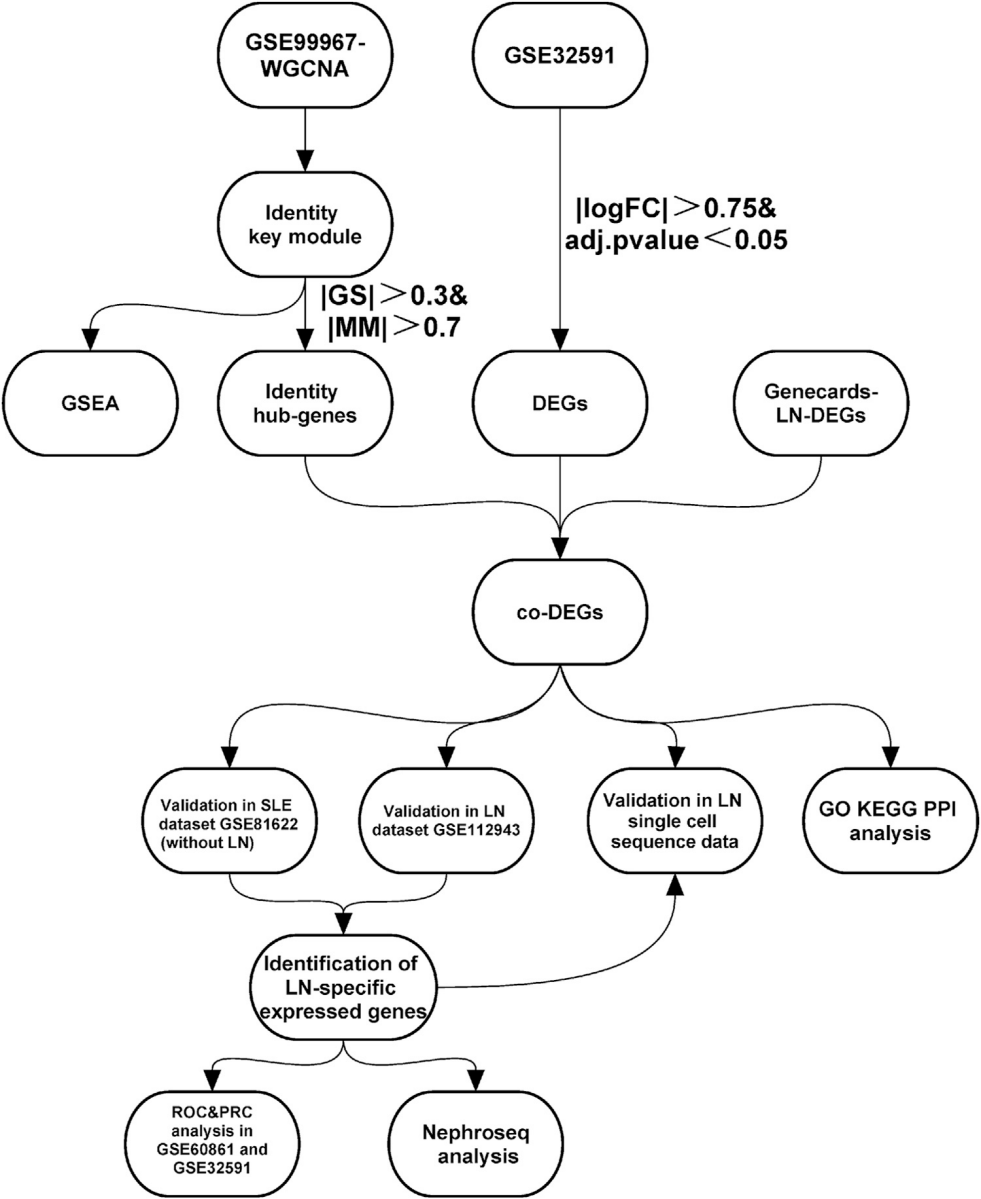
**|** Study workflow. Abbreviations: Systemic lupus erythematosus (SLE); Lupus nephritis (LN); Weighted gene co-expression network analysis (WGCNA); Gene set enrichment analysis (GSEA); Differentially-expressed genes (DEGs); Gene ontology (GO); Kyoto encyclopedia of genes and genomes (KEGG); Protein-Protein interaction (PPI); Receiver operating characteristic curve (ROC); Precision-Recall curve (PRC).

We searched the GEO database (https://www.ncbi.nlm.nih.gov/geo/) (Barrett et al., 2013) for human SLE- and LN-related expression profiles using the keywords “lupus nephritis” and “systemic lupus erythematosus.” GSE32591 is based on the GPL14663 platform, which includes 29 normal kidney biopsy and 64 LN kidney biopsy samples (Berthier et al., 2012). We extracted data from 17 normal blood samples and 29 peripheral blood samples from SLE patients with lupus nephritis from the GSE99967 dataset (based on the GPL21970 platform) for subsequent analysis (Wither et al., 2018). In addition, we extracted data from seven normal and 14 lupus nephritis samples from the GSE112943 dataset (built on the GPL10558 platform). GSE81622 is based on the GPL10558 platform and includes 25 normal and 15 SLE samples of patients without LN (Zhu et al., 2016). The GSE60681 dataset based on the GPL13497 platform includes data from 11 patients with LN and from 37 control samples in stable phase (Magnusson et al., 2017). Finally, we also searched the Genecards database (https://www.genecards.org/) using the keyword “lupus nephritis” to identify differential genes associated with LN. Figure 1 illustrates the specific applications used and the workflow for all data in this study.

### Data Pre-Processing

We used the Perl language to process the original matrix of GSE32591, GSE99967, GSE112943, GSE81622, and GSE60681. The probe IDs were converted to gene symbols, and empty probes were removed based on the annotation information contained in each platform file. When multiple probes matched the same gene, the average expression value was used to determine the gene’s expression level. The Perl script we used to pre-process is detailed in Supplementary Data S1.

### Weighted Gene Co-expression Network Analysis

We used the R language package WGCNA (Langfelder and Horvath, 2008) to evaluate the GSE99967 expression matrix. We extracted the LN grouping, SLEDIA-2K score, age, and gender data from the original set as input data for the WGCNA. The sample clustering dendrogram was constructed with the hcluster function, and the TOM matrix was constructed using the pickSoftThreshold function to determine the optimal soft threshold. We used candidate power values (1–30) to determine the average connectivity and independence of various modules. Dynamic shear trees were used to identify gene modules. Next, we measured the association between modules and sample traits using gene significance values (GS) and module membership values (MM), and key modules were identified. We set |GS| to >0.3 and |MM| to >0.7 to filter hub genes in accordance with the official WGCNA guidelines and prior application examples to obtain the most relevant genes to the traits in the key module (Langfelder and Horvath, 2008; Tang et al., 2018).

### Identification of Co-Differentially-Expressed Genes

The annotated GSE32591 expression matrix was analyzed for differentially-expressed genes (DEGs) using the R limma package (http://www.bioconductor.org/packages/release/bioc/html/limma.html) (Smyth, 2004) setting the |logFC| to >0.75 and the adjusted *p*-value to <0.05 as the criterion. The DEGs were then intersected with the module hub genes identified using WGCNA in the GSE99967 set and the LN-related differential genes used in the Genecards database to identify co-expressed LN differential genes (co-DEGs) across multiple source datasets.

### Gene Set Enrichment Analysis

We performed a gene set enrichment analysis (GSEA) (Subramanian et al., 2005) using the KEGG and REACTOME gene sets in the GSEA C2 dataset (c2.cp.kegg.v7.4.symbols.gmt c2.cp.reactome.v7.4.symbols.gmt), the GO gene set in the C5 dataset (c5.go.bp.v7.4.symbols.gmt, c5.go.cc.v7.4.symbols.gmt, c5.go.mf.v7.4.symbols.gmt), and the hallmarker gene set h.all.v7.4.symbols.gmt). |NES| > 1, NOM *p*-value < 0.05, FDR q-value < 0.25 were set as the screening criteria for enrichment pathways. The results were visualized using the OmicShare (http://www.omicshare.com) cloud platform tool.

### Gene Ontology, Kyoto Encyclopedia of Genes and Genomes Enrichment Analysis and Protein-Protein Interaction Network Construction

We performed gene ontology (GO) (Ashburner et al., 2000) and Kyoto encyclopedia of genes and genomes (KEGG) (http://www.genome.jp/kegg/) (Kanehisa and Goto, 2000) enrichment analyses of co-DEGs using the ClueGo (Bindea et al., 2009) and the Cluepedia (Bindea et al., 2013) plugins within the CytoScape software (V 3.7.2, http://www.cytoscape.org/) (Cline et al., 2007), setting *p* to <0.05 to screen the results and construct the target-pathway network. The co-DEGs were submitted to the STRING database (https://www.string-db.org/) (Szklarczyk et al., 2015) to evaluate the interaction between co-DEGs from the protein level; we obtained protein interaction network co-DEGs by setting the confidence level to 0.4.

### Validation of Co-Differentially-expressed genes

We validated the expression levels of co-DEGs in the GSE112943 LN expression profile dataset and the GSE81622 SLE expression profile dataset without lupus nephritis to verify that co-DEGs have similar expression profiles in different LN datasets and to verify whether the co-DEGs were specific to LN. Significance tests were performed using the Wilcoxon–Mann–Whitney test, with results visualized using the ggplot2 package (Villanueva and Chen, 2019). We used the PRROC package (Grau et al., 2015) to examine the co-DEGs’ diagnostic efficacy by performing Receiver Operating Characteristic (ROC) and Precision-Recall curves (PRC) analyses in the GSE60681 dataset. We validated the distribution of co-DEGs in the published LN single-cell transcriptome sequencing database (https://singlecell.broadinstitute.org/) to explore co-DEGs’ distribution in LN immune cells) (Arazi et al., 2019). Finally, we analyzed the association between co-DEGs and clinical features using the Nephroseq database (http://v5.nephroseq.org/) (Zheng et al., 2017). A scatter plot was constructed after calculating the Pearson correlation coefficient between co-DEGs and glomerular filtration rates (GFRs). The Kruskal–Wallis test was used to test the significance of co-DEGs and lupus pathological staging.

## Results

### Weighted Gene Co-Expression Network Analysis Identifies Key Lupus Nephritis Genes

**FIGURE 2:**
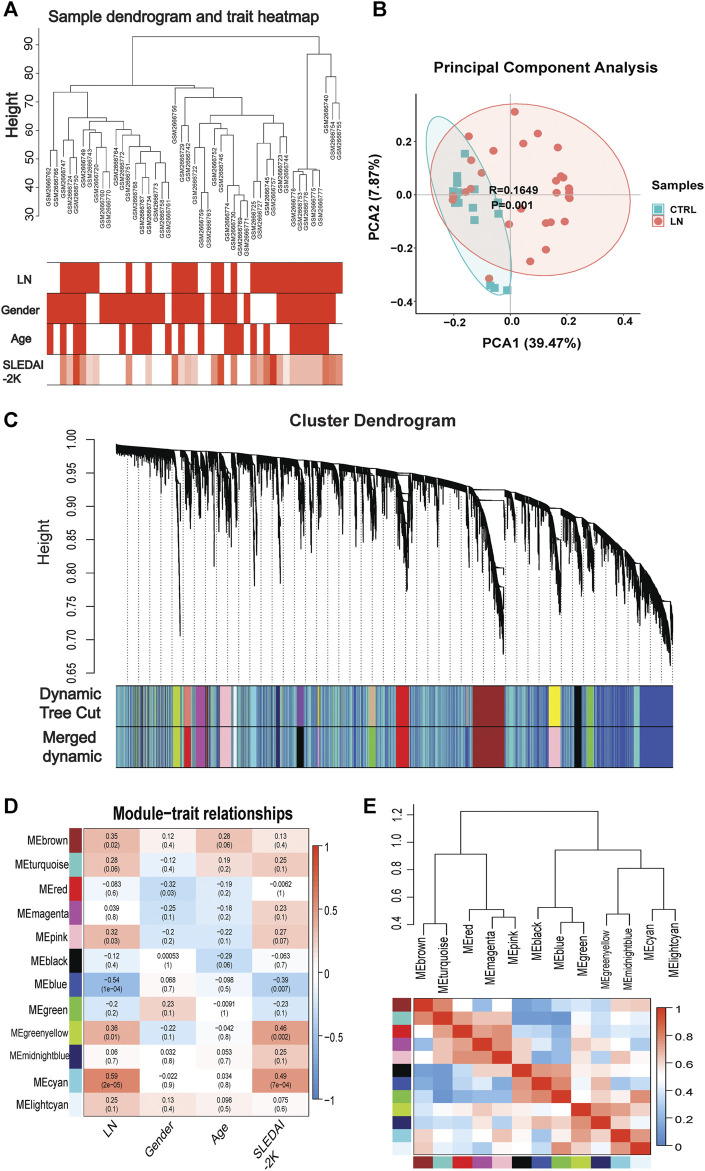
**|** WGCNA analysis in GSE99968. **(A)** Sample-trait clustering heatmap. **(B)** Principal component analysis (PCA) shows the dimensionality reduction distribution of control and LN sets. **(C)** Dynamic shearing tree merging similar module genes. **(D)** Module-trait correlation heatmap. **(E)** Module-module clustering tree and correlation heatmap.

Genes of interest can be identified by combining gene and clinical trait data and dividing the gene co-expression network of complex biological processes into several highly correlated signature modules that can detect the genes that perform critical functions. As shown by the hierarchical clustering in Figure 2A, potential differences between control and LN clusters exist between the different clinical phenotypes. Our principal component analysis (PCA) results show the dimensionality reduction distribution of control and LN sets (Figure 2B). We used WGNCA to analyze the GSE99967 expression matrix, with the shear height of the function hcluster set to 100 and an outlier sample GSM266765 excluded (Supplementary Figure S1). We calculated the pickSoftThreshold parameter to determine the optimal soft threshold, which is 4 (Supplementary Figure S2). The dynamic shear tree’s merged shear height was 0.25 for module identification and module merging (Figure 2C). The minimum number of genes in each network module was set to 120, resulting in a total of 12 gene modules. The most strongly correlated positive and negative modules were chosen as critical modules for the pathogenesis of the LN and SLEDAI-2K traits. Our results indicate that the blue module was significantly negatively correlated with the LN trait, while the cyan module was significantly positively correlated with the SLEDAI-2K trait (Figures 2D,E). These two modules were identified as critical modules, and when |GS|>0.3 and |MM|>0.7 were used to screen for essential genes, we found 2255 genes in the LN trait and 1,388 genes in the SLEDAI-2K trait.

### Gene Set Enrichment Analysis Enrichment Analysis

**FIGURE 3:**
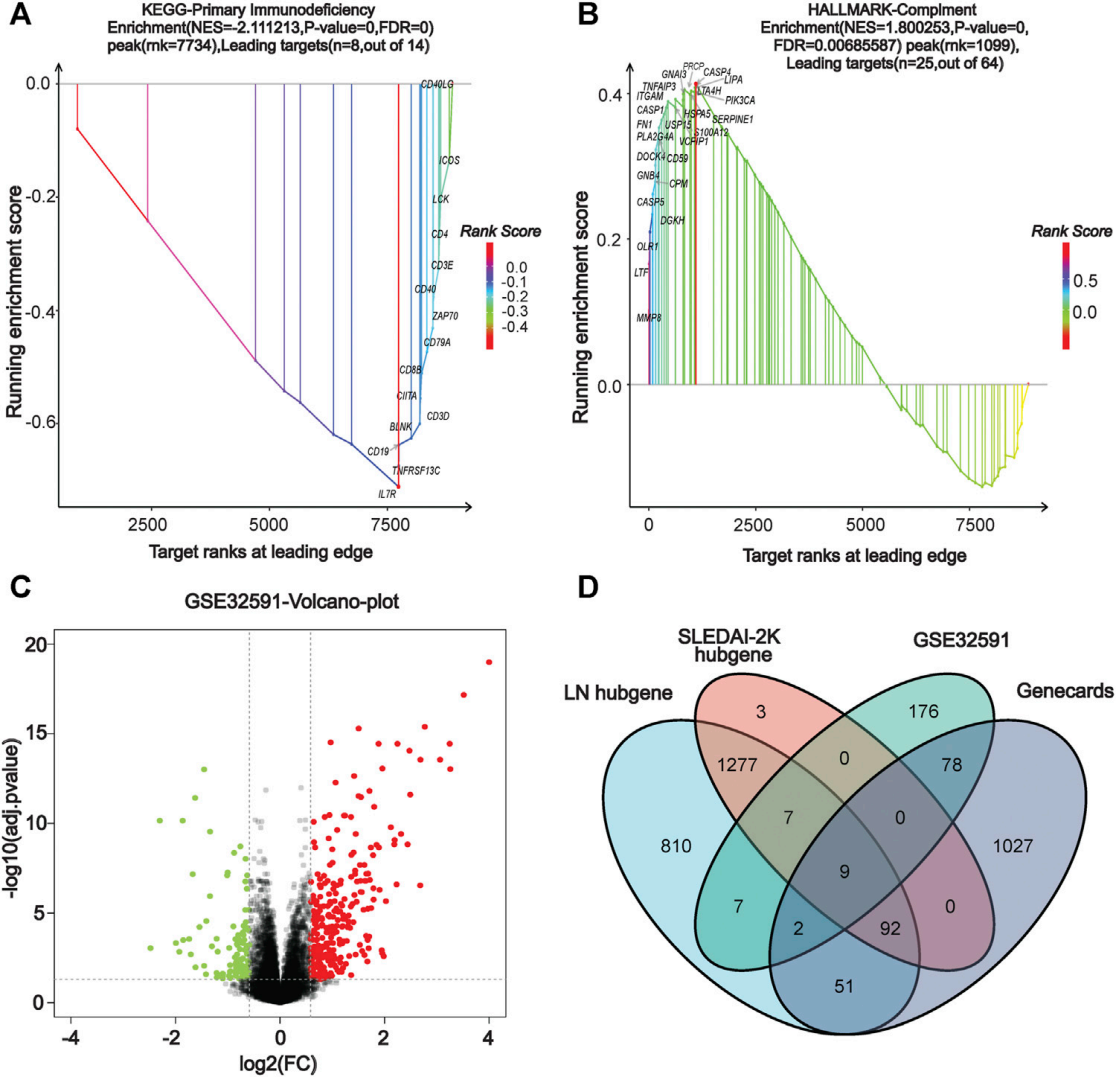
**|** GSEA enrichment analysis and identification of co-DEGs. **(A)** KEGG dataset enrichment results. **(B)** HALLMARKER dataset enrichment results. **(C)** Volcano map of GSE32591 differentially-expressed genes. **(D)** Venn diagram screening for co-differentially-expressed genes.

We performed a comprehensive enrichment analysis of the screened blue and cyan modules to discover the functions or pathways associated with LN using the GSEA software and exploring the functions and pathways of the key modules. Our results show that the key modules were mainly enriched for GO entries (including antimicrobial humoral response, defense response to fungus), and KEGG pathways (including cell cycle, P53 signaling pathway, systemic lupus erythematosus, and primary immunodeficiency). The hallmark entries included G2M checkpoint, mitotic spindle, and REACTOME pathways including the RNA pol-I promoter opening and meiotic recombination pathways (Supplementary Figure S3). Figures 3A,B present two GSEA pathways that are highly associated with lupus pathogenesis. The complete GSEA enrichment results are presented in Supplementary Table S1.

### Identification of Differentially-Expressed Genes and Screening of Co-Differentially-Expressed Genes

In our screen for critical LN genes, we used a variety of methods to obtain differentially-expressed LN genes. The differential genes in GSE32591 were screened using the R limma package, setting the |logFC| to >0.75 and the adjusted *p*-value to <0.05 as criteria, yielding a total of 216 up-regulated and 63 down-regulated differential genes. The volcano plot in Figure 3C depicts the distribution of DEGs. Additionally, we entered the keyword “lupus nephritis” to search the Genecards database for LN-related differential genes and found 1,248 genes (Supplementary Table S2). After using the VennDiagram package (Chen and Boutros, 2011) to intersect the critical genes of WGCNA, the DEGs of GSE32591, and the LN-related genes in the Genecards database, we identified nine co-expressed differential genes in multiple source datasets: *TLR2*, *LTF*, *IL10RA*, *IRF8*, *CD163*, *HLA-DMA*, *HLA-DRA*, *HLA-DPA1*, and *HLA-DPB1* (Figure 3D).

### Co-Differentially-Expressed Genes Gene Ontology and Kyoto Encyclopedia of Genes and Genomes Enrichment Analysis and Protein-Protein Interaction Network Construction

**FIGURE 4:**
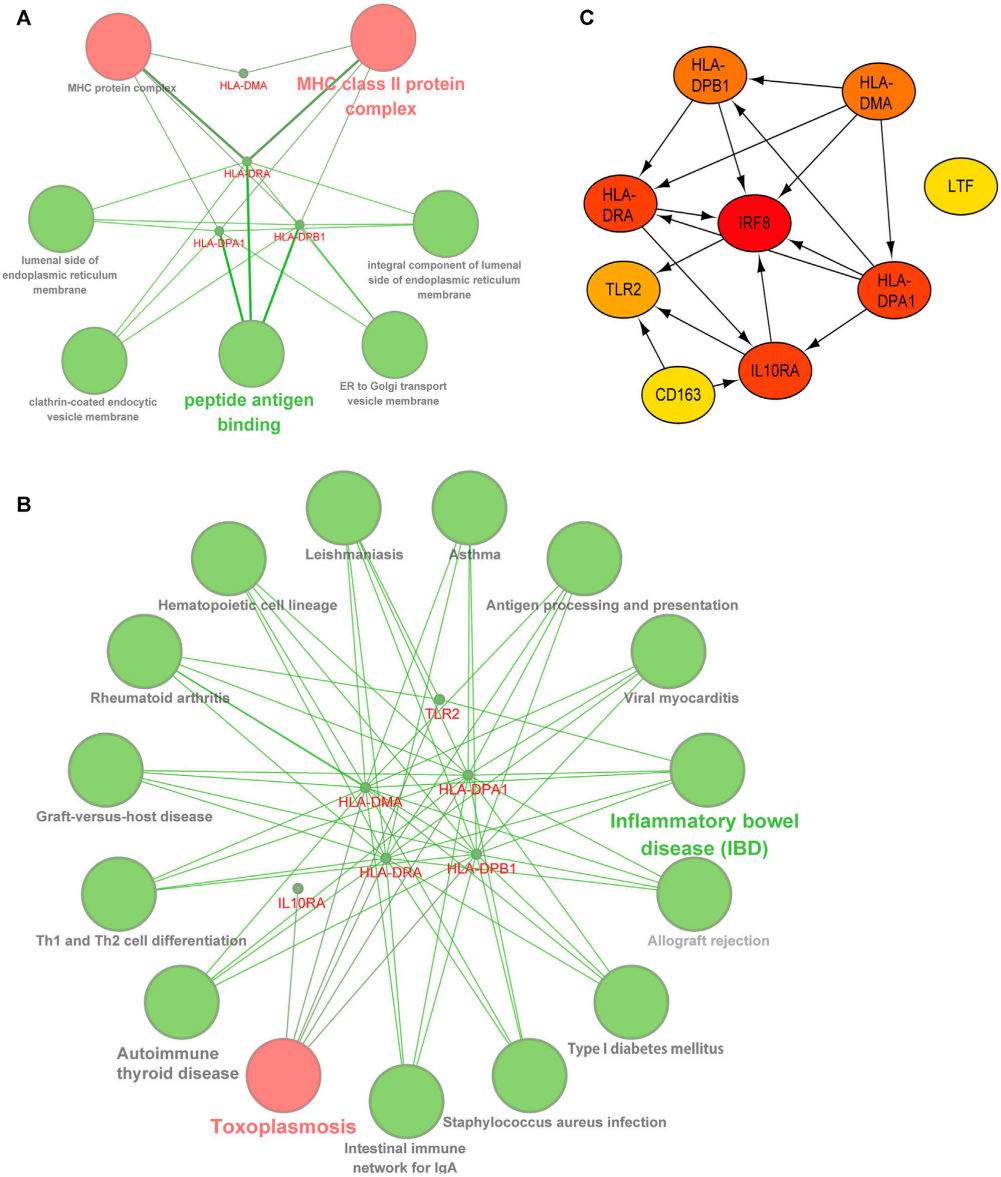
**|** Functional and pathway analysis of co-DEGs. **(A)** GO enrichment analysis of co-DEGs. **(B)** KEGG enrichment analysis of co-DEGs. **(C)** Protein-protein interaction network of co-DEGs, color shades represent the degree size calculated with the cytoHubba plugin.

We deduced the specific functions and pathways of co-DEGs via GO and KEGG enrichment analyses. Additionally, the PPI network results uncovered intrinsic co-DEG connections. The ClueGo plugin in Cytoscape software can show a network of the association between pathways and the enrichment of genes among them. We imported co-DEGs into ClueGo for analysis, and the results show that the main GO entries enriched by co-DEGs included those for the MHC and MHC class II protein complexes (Figure 4A). The KEGG pathways involved include those for toxoplasmosis, inflammatory bowel disease, and others (Figure 4B). Our STRING database results showed the protein interaction associations of co-DEGs; we imported the results into CytoScape software to calculate the degree values between the networks (visualized using the cytoHubba plugin). *IRF8* was the hub gene in the network (Figure 4C).

### Validation of Co-Differentially-Expressed Genes in Other Lupus Nephritis and Systemic lupus erythematosus Datasets

**FIGURE 5:**
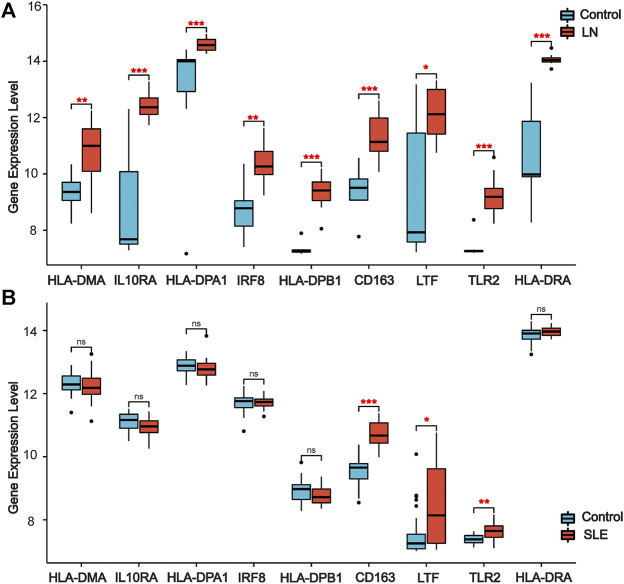
**|** Validation of co-DEGs in additional LN and SLE datasets. **(A)** Expression of co-DEGs in the additional LN dataset GSE112943. **(B)** Expression of co-DEGs in the GSE81622 SLE dataset. (*p < 0.05, **p < 0.01, ***p < 0.001)

We used other LN and SLE expression profile data to validate the expression of co-DEGs in different datasets. Our results indicated that all co-DEGs were differentially expressed between control and LN groups in the LN GSE112943 set (Figure 5A). The expression levels of *IRF8*, *IL10RA*, *HLA-DPA1*, *HLA-DPB1*, *HLA-DMA*, and *HLA-DRA* were not significantly different between the control and SLE groups (without LN) in the GSE81622 expression profile data. (Figure 5B). Therefore, these six genes highly expressed in LN samples indicated that they may serve as novel biomarkers for the disease.

### Validation of Co-Differentially-Expressed Genes’ Distribution in Single-Cell Datasets

**FIGURE 6:**
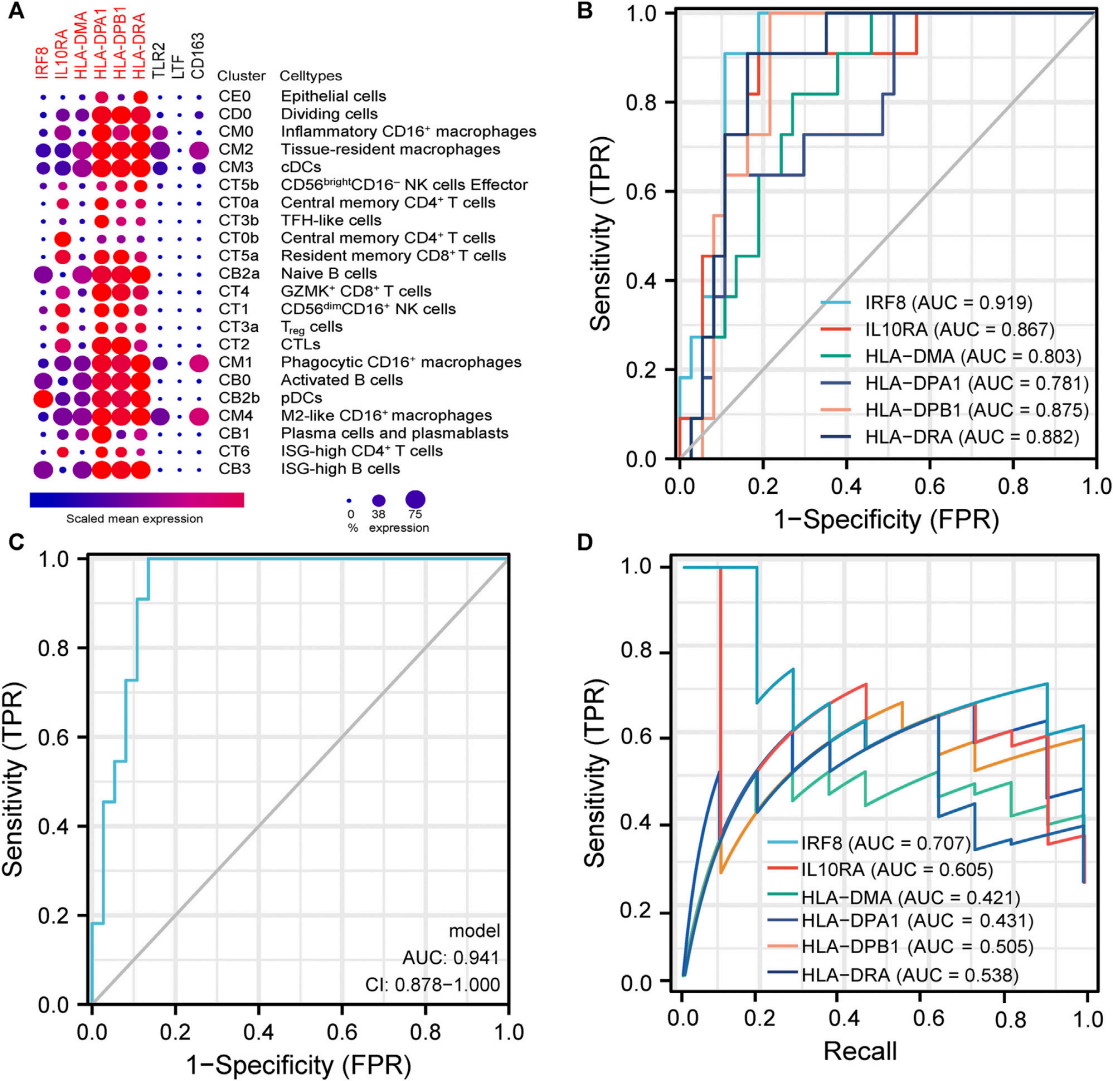
**|** Analysis of immune cell distribution and diagnostic efficacy of LN biomarkers **(A)**. Expression of co-DEGs in different immune cell types in the single cell LN sequencing database. **(B)** ROC curves of six differentially-expressed genes specific to LN in GSE60861. **(C)** ROC curve with optimal minimal gene set (*IRF8*, *IL10RA*, *HLA-DMA*, *HLA-DPA1*) in GSE60861. **(D)** Precision-Recall Curves of six differentially-expressed genes specific to LN in GSE60861.

Due to the close association between LN pathogenesis and immune disorders, we investigated the distribution of co-DEGs in immune cells using the published LN single-cell sequencing database. We found that among the six co-DEGs explicitly expressed in LN, HLA-related genes were overexpressed in a variety of macrophage subtypes (inflammatory CD16^+^ macrophages, tissue-resident macrophages, phagocytic CD16^+^ macrophages) and B cells (naive B cells, ISG-high B cells, activated B cells). However, *IRF8* and *IL10RA* were expressed at a relatively low level in macrophages and B cells. Additionally, three other co-DEGs, *TLR2*, *LTF*, and *CD163*, were only partially expressed in macrophages (tissue-resident macrophages, phagocytic CD16^+^ macrophages, M2-like CD16^+^macrophages) (Figure 6A).

### Diagnostic and Prognostic Analysis of Six Co-Differentially-Expressed Genes

**FIGURE 7:**
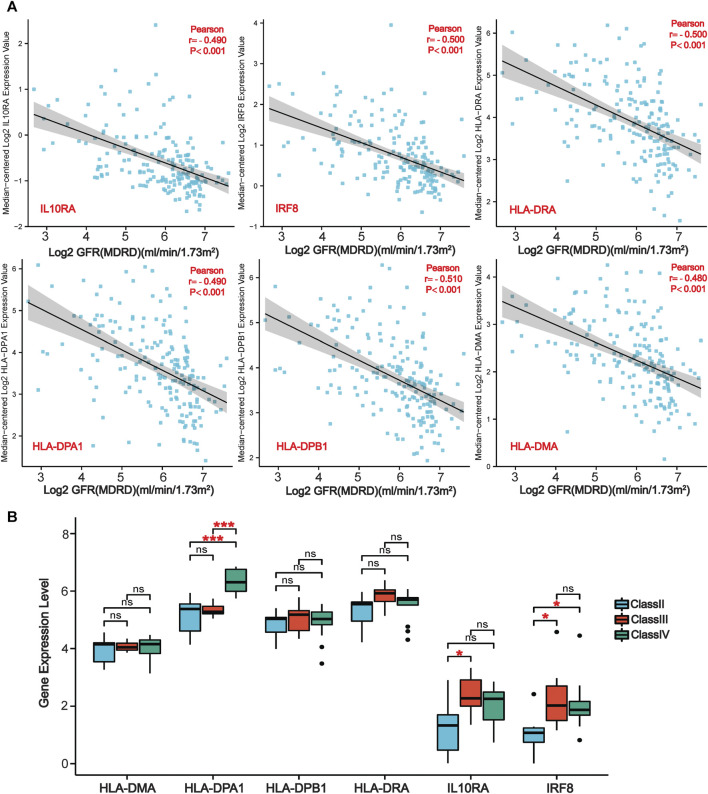
**|** Association of co-DEGs with LN clinical traits in the Nephroseq database. **(A)** Correlation analysis of six co-DEGs with the glomerular filtration rate of patients (GFR). **(B)** Variations of six co-DEGs in samples from patients with different pathological staging of lupus nephritis. (*p < 0.05, ***p < 0.001).

We further evaluated the six LN-specific co-DEGs as diagnostic biomarkers by using ROC curves and a PRC analysis in a new LN dataset GSE60681 to assess the sensitivity and specificity of co-DEGs for LN diagnosis. As shown in Figure 6B, the ROC-AUC values for all six genes were greater than 0.78 (*IRF8*, 0.919; *HLA-DPB1*, 0.875; *HLA-DRA*, 0.882; *IL10RA*, 0.867; *HLA-DMA*, 0.803; and *HLA-DPA1*, 0.781) suggesting that these six genes have good diagnostic efficacy as LN markers. To obtain the minimum set of genes with the greatest predictive power, we tested combinations of the six genes; we found that the combination of *IRF8*, *IL10RA*, *HLA-DMA*, and *HLA-DPA1* had the greatest AUC value (0.94) among the 15 tested combinations (Figure 6C). We also performed a PRC analysis to compensate for the imbalance of the selected samples, and our results showed that *IRF8* (PR-AUC, 0.707) and *IL10RA* (PR-AUC, 0.605) retained a good diagnostic sensitivity despite the unevenness of the samples (Figure 6D). However, considering that the results of this method are affected by the number of positive and negative samples, analysis using a dataset with a different number of imbalanced samples would allow for a more comprehensive assessment of the results (that is, both ROC-AUC and PR-AUC showed good diagnostic efficacy when analyzed using the GSE32591 dataset; please see Supplementary Figures S4, S5). Thus, we believe that all six genes have good diagnostic efficacy after combining the ROC and PRC results from different datasets. To assess the association between co-DEGs and LN prognostic factors, we validated the association between co-DEGs and clinical traits in LN samples from the Nephroseq database. Our findings indicate that a high expression of different co-DEGs was correlated with a low glomerular filtration rate in kidney disease samples (*IL10RA*, *HLA-DPA1*, r = −0.490, *p* < 0.001; *IRF8*, *HLA-DRA*, r = −0.500, *p* < 0.001; *HLA-DPB1*, r = −0.510, *p* < 0.001; *HLA-DMA*, r = −0.480, *p* < 0.001) (Figure 7A). Further analysis revealed that *HLA-DPA1*, *IL10RA*, and *IRF8* were differentially expressed in different pathological staging samples of lupus nephritis (Figure 7B).

## Disscussion

We identified nine differential genes that were simultaneously significant in datasets from multiple sources. Six of the genes are LN-specific and are associated with a poor prognosis, and their good diagnostic efficacy suggests that these genes can serve as novel LN biomarkers. In addition, a comprehensive functional and pathway enrichment approach revealed that the biological mechanisms mediating LN development are interrelated. A single DEG identification approach may miss some DEGs, but not those that are significantly differentially expressed; thus, the co-expressed genes identified in this study by integrating multiple approaches may be critical for elucidating the pathogenesis of LN.

The SLEDIA-2K score is a tool for assessing the severity of SLE (Touma et al., 2018). Our WGCNA analysis revealed that the critical modules derived from the LN trait are consistent with those derived from the SLEDIA trait, indicating that the module genes obtained are genuinely involved in the pathogenesis of LN. LN is primarily caused by an antigen-antibody complex immune response that results in large amounts of autoantibodies in the intrarenal space (Qiu et al., 2019). These atypical antigen-antibody reactions result in vascular damage, abnormal complement activation, complex deposition, and an imbalance of the oxidative/antioxidant and cytokine systems (Davidson et al., 2019; Anders et al., 2020). Our results on the function and pathways of critical gene enrichments confirm these mechanisms (Supplementary Table S1).

Among the LN-specific DEGs identified, *HLA-DMA*, *HLA-DPA1*, *HLA-DPB1*, and *HLA-DRA* all belong to the human major histocompatibility complex (MHC) and the HLA class II region, alternatively referred to as the HLA-D region. Most genes in this region are involved in immune responses and are classified into several subregions (*HLA-DR*, *DQ*, *DP*, *DO*, and *DM*) (Wieczorek et al., 2017; Wang et al., 2019). SLE has been linked to polymorphisms in the HLA-D region that vary by race and geographical region. Alleles at the same HLA locus, which may differ structurally by a few nucleotides, can result in completely different disease susceptibility or resistance profiles (Xue et al., 2018). This explains the differential expression of the four HLA genes we identified in the SLE and LN datasets, with genetic polymorphisms resulting in systemic and local pathological changes (Xu et al., 2017). *IRF8* is a member of the interferon regulatory factor (IRF) family that regulates the signaling pathway for Toll-like receptors (Salem et al., 2020). Alternatively, *IRF8* regulates Th cell differentiation, thereby regulating immune cell development and inhibiting tumor cell growth. Silencing the *IRF8* gene in SLE mice has been suggested to inhibit DC-mediated activation of NF-κβ or MAPKs, thereby impairing type I interferon induction (Salem et al., 2020). IL-10 is a multifunctional cytokine derived from multicellular organisms that functions only when bound to a specific receptor (Moore et al., 2001). IL-10 interacts with *IL10RA* and delivers excitatory or inhibitory signals to cells via the JAK-STAT signal transduction pathway (Geginat et al., 2019). Increased IL-10 expression in LN kidney tissues is associated with an increase in macrophage infiltration and is highly correlated with the severity of kidney damage (Saraiva et al., 2020). Additionally, our results suggest that co-DEGs are differentially expressed primarily in macrophages and B cells, a finding consistent with the previous view that different types of macrophages and B cells play more important roles in LN (through complex interactions) than T cells (Arazi et al., 2019).

In summary, we identified several valuable biomarkers associated with the diagnosis and prognosis of lupus nephritis. These biomarkers are involved in a variety of different molecular pathways expressed in various immune cells. However, additional research is necessary to determine the association between specific HLA alleles and LN because of the presence of HLA gene polymorphisms. In addition, the lack of a definitive experimental validation represents a limitation of our study. We will focus on establishing more conclusive and robust evidence for the validity of these identified co-DEGs as novel biomarkers in subsequent studies.

## Conclusion

We found nine differentially expressed genes closely associated with LN diagnosis and prognosis by integrating multiple DEG identification methods. Next, we identified six biomarkers that may be LN-specific by expression validation in LN and SLE datasets. A comprehensive gene enrichment analysis revealed that the molecular mechanisms associated with LN pathogenesis are linked to multiple critical immune pathways. Finally, we explored the distribution of co-DEGs in LN immune cells by analyzing data from a single-cell transcriptome sequencing database of LN. Our prioritized biomarkers should be helpful for the diagnosis and prognosis of LN and they should deepen our understanding of its pathogenesis.

## Data Availability

The datasets presented in this study can be found in online repositories. The names of the repository/repositories and accession number(s) can be found in the article/Supplementary Material.
