# Supplementary material for: Prioritization of Diagnostic and Prognostic Biomarkers for Lupus Nephritis Based on Integrated Bioinformatics Analyses
Source: Front Bioeng Biotechnol. 2021 Oct 8;9:717234. doi: 10.3389/fbioe.2021.717234 (PMC8531593; doi:10.3389/fbioe.2021.717234)
Supplement: Supplementary file 3 [file Table3.DOCX]

Supplementary Material


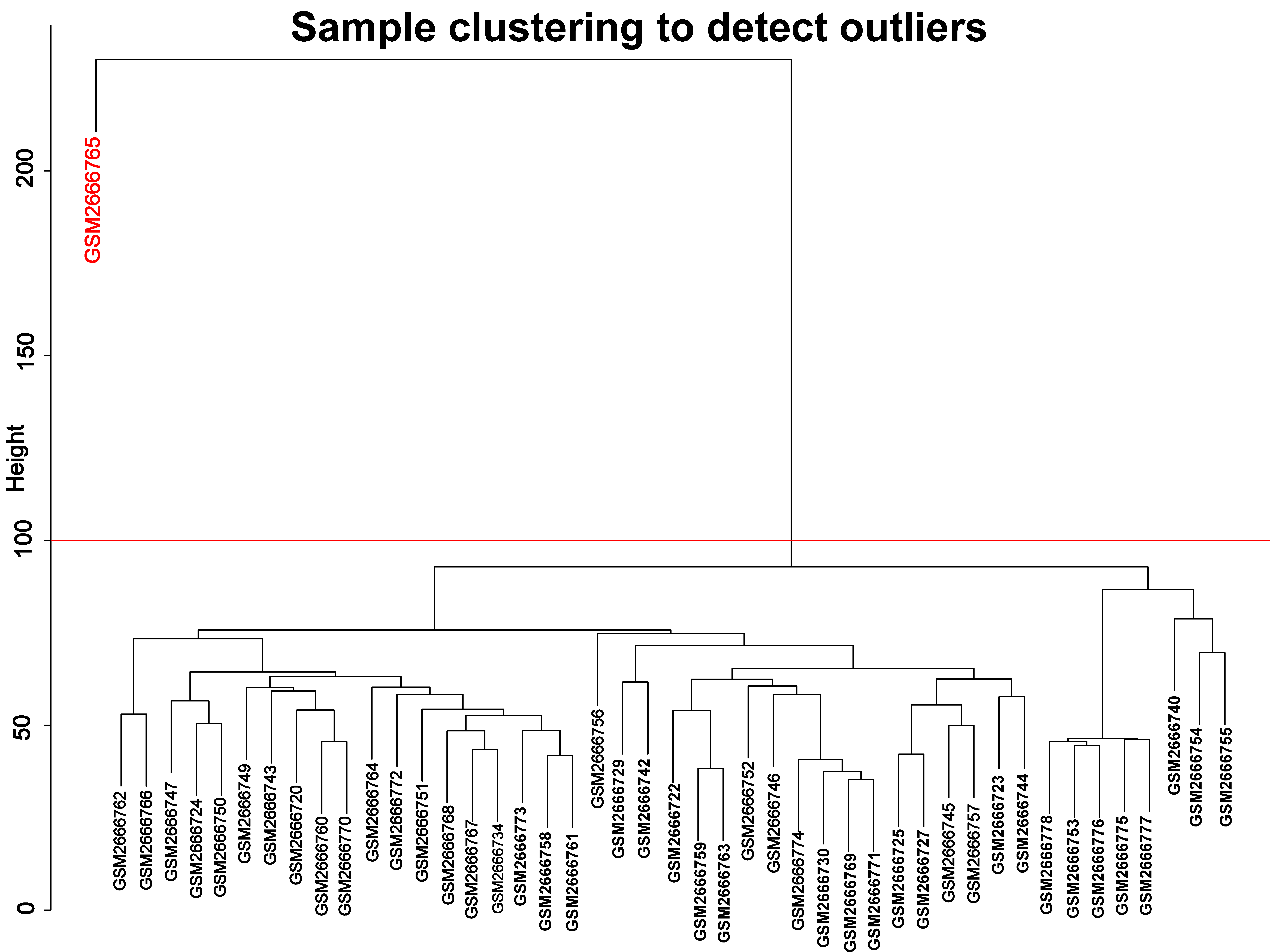


**Supplementary Figure 1.** WGCNA detects the outlier sample. The shear height of the function hcluster to 100 was set to exclude outlier samples, and an outlier sample GSM266765 was excluded

**
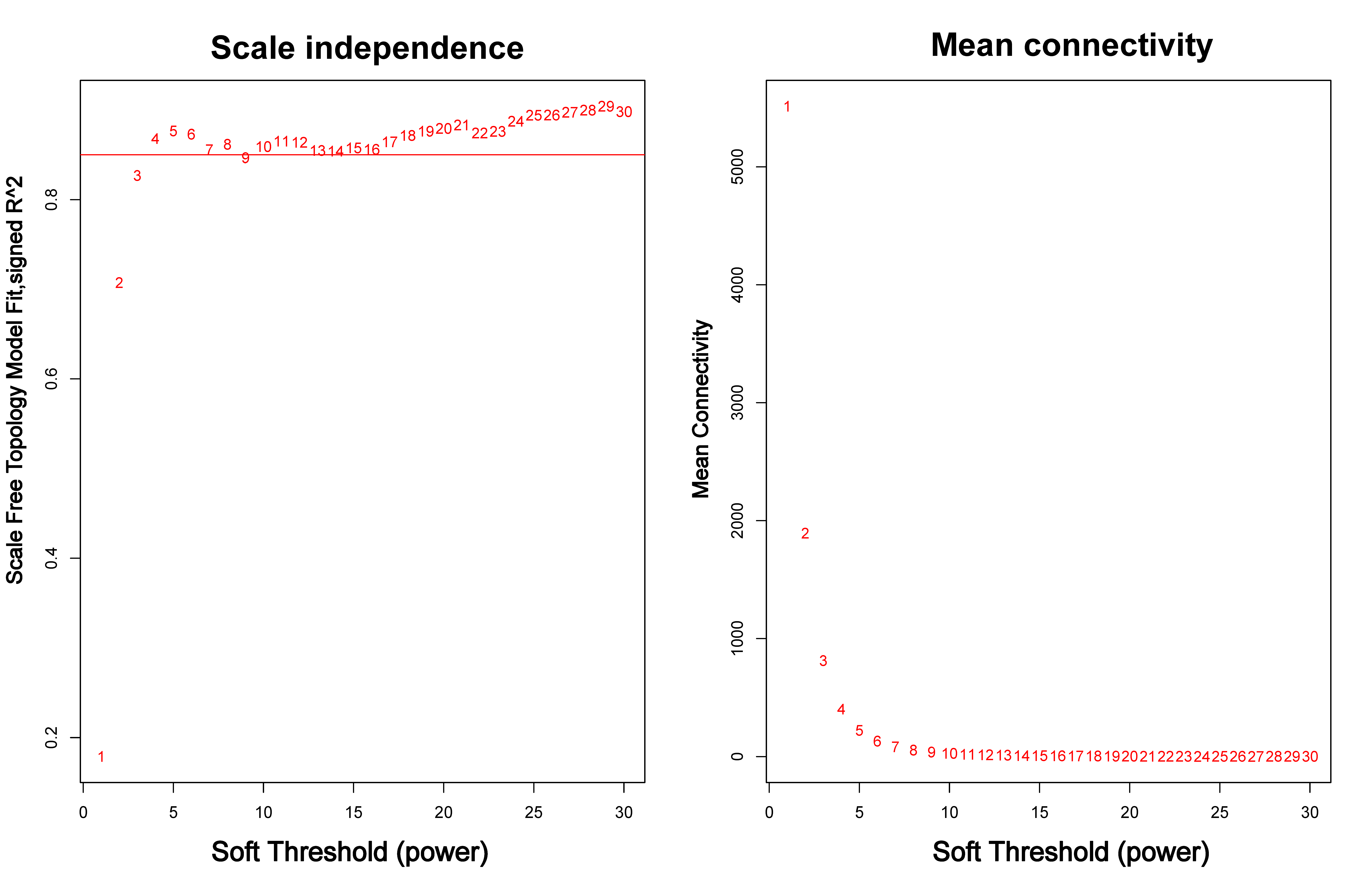
**

**Supplementary Figure 2.** WGCNA selects optimal soft threshold powers. Scale-free matching index for different soft threshold powers (left). Average connectivity at different soft threshold powers (right).


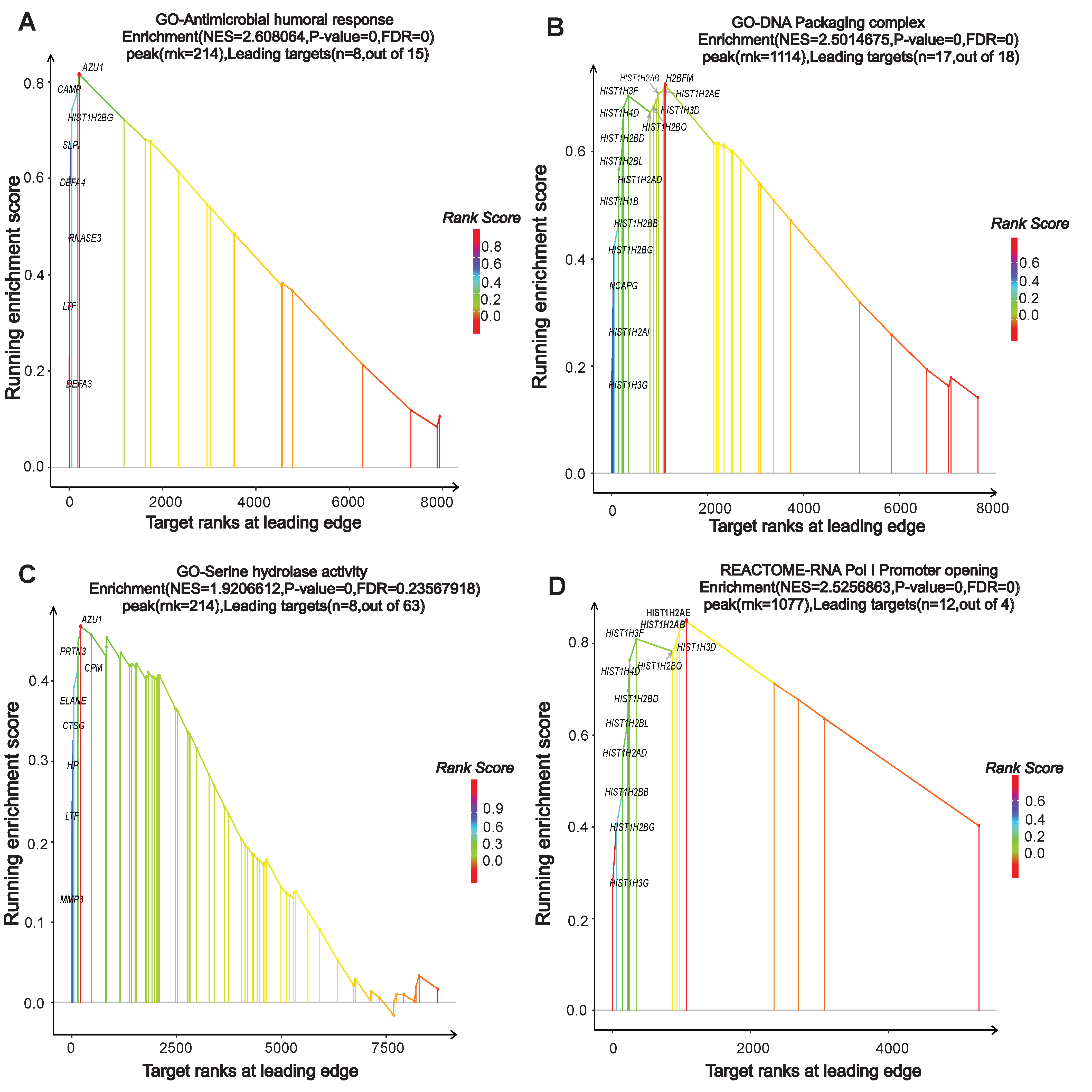


**Supplementary Figure 3.** GSEA enrichment analysis for key modules (Representative Results). **(A)** Results of biological process enrichment in GO dataset. **(B)** Results of cellular component enrichment in GO dataset. **(C)** Molecular function enrichment results in GO dataset. **(D)** REACTOME dataset enrichment results.


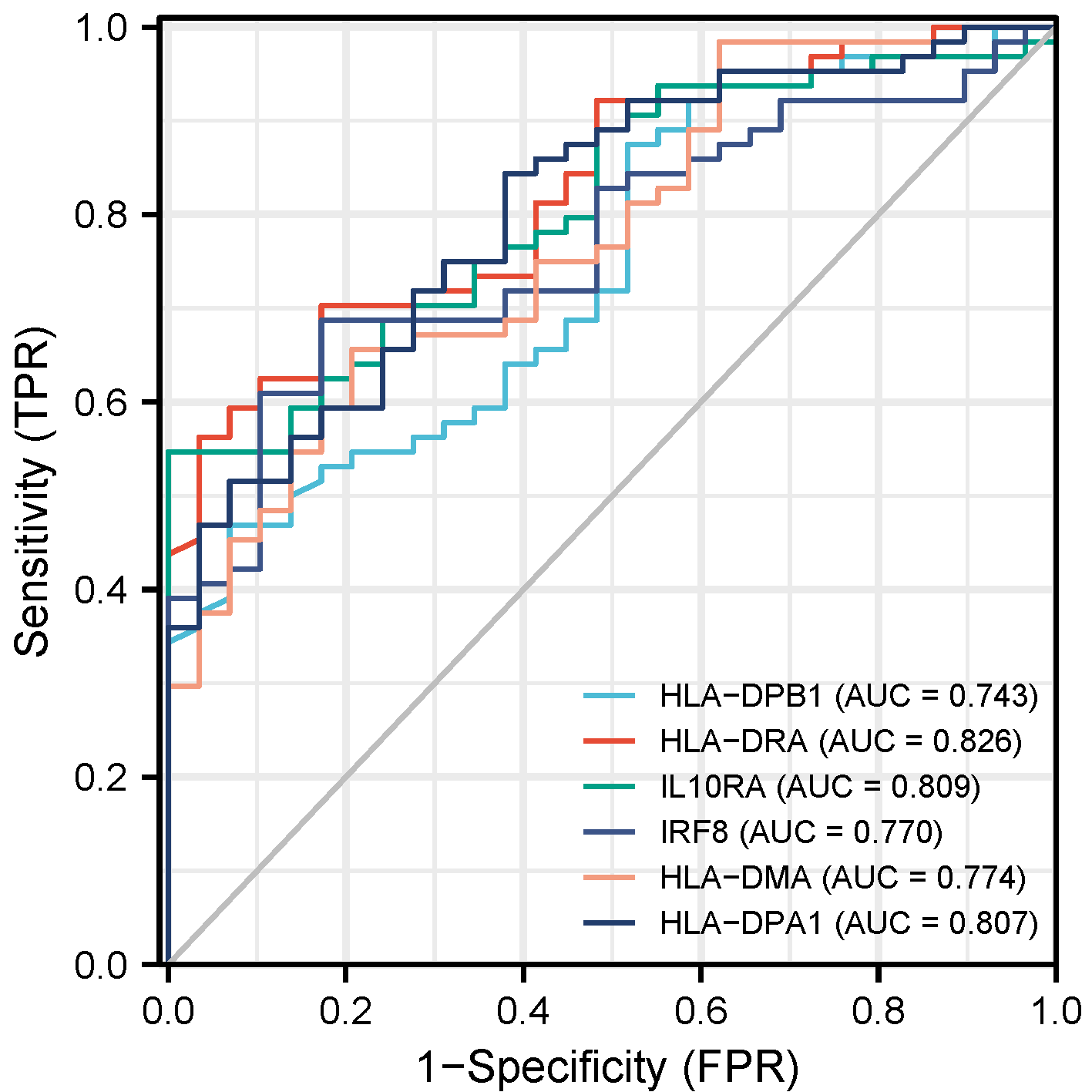


**Supplementary Figure 4.** ROC curves of 6 differentially expressed genes specific to LN in GSE32591.


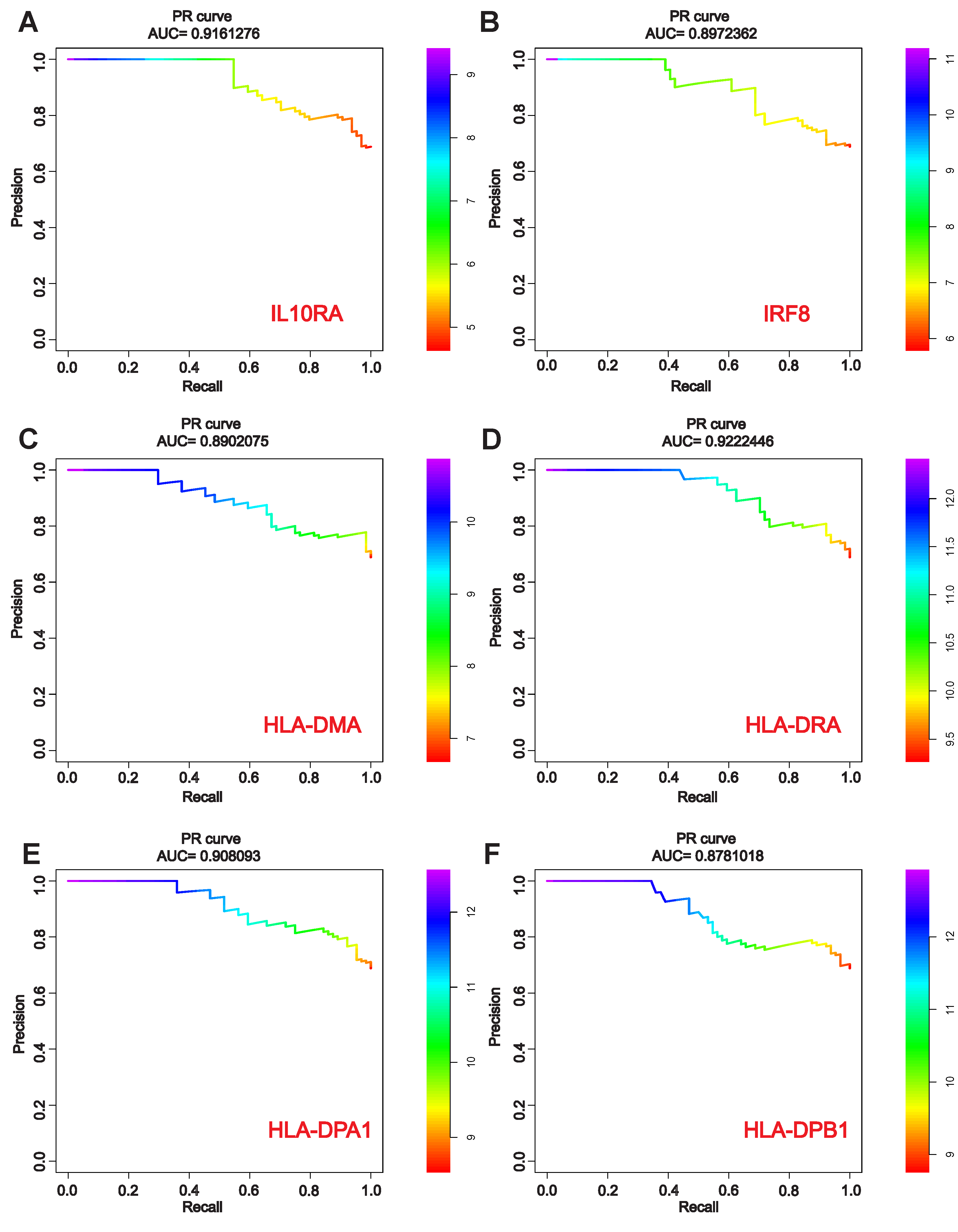


**Supplementary Figure 5.** Precision-Recall Curve (PRC) of 6 differentially expressed genes specific to LN in GSE32591. **(A-F)** IL10RA, IRF8, HLA-DMA, HLA-DRA, HLA-DPA1, HLA-DPB1.
